# Burden of Pancreatitis in the United States from 1990 to 2023: Findings from the Global Burden of Disease Study 2023

**DOI:** 10.3390/jcm15197497

**Published:** 2026-09-26

**Authors:** Murali Mohan Rama Krishna Reddy, Anish Chopra, Ganesh Aswath, Vivek Kesar, Joydeep Chakraborty, Abdul Haseeb, Jassimran Singh, Kailash Bajaj, Aaysha Kapila, Hassam Ali, Arkadeep Dhali, Dushyant Singh Dahiya

**Affiliations:** 1 Department of Internal Medicine, Northwest Health—Porter, Valparaiso, IN 46383, USA; 2 Department of Gastroenterology and Interventional Endoscopy, Northwest Health—Porter, Valparaiso, IN 46383, USA; 3 Department of Internal Medicine, Division of Gastroenterology and Hepatology, State University of New York—Upstate Medical University, Syracuse, NY 13210, USA; 4 Division of Gastroenterology and Hepatology, Stony Brook Medicine, New York, NY 11794, USA; 5 Division of Gastroenterology and Transplant Hepatology, University of Mississippi Medical Center, Jackson, MS 39216, USA; 6 Department of Gastroenterology and Interventional Endoscopy, Advocate Lutheran General Hospital, Park Ridge, IL 60068, USA; 7 Department of Gastroenterology, Hepatology and Motility, University of Kansas Medical Center, Kansas City, KS 66160, USA; 8 Department of Gastroenterology, Hepatology and Nutrition, East Carolina University/Brody School of Medicine, Greenville, NC 27834, USA; 9 School of Medicine, Dentistry and Biomedical Sciences, Queen’s University Belfast, Belfast BT7 1 NN, UK; 10 Division of Gastroenterology and Hepatology, Turkey Creek Medical Center, Tennova Healthcare, Knoxville, TN 37849, USA; dush.dahiya@gmail.com

**Keywords:** pancreatitis, burden of pancreatitis, epidemiology of pancreatitis

## Abstract

**Objectives:** Pancreatitis is the third leading cause of gastrointestinal disease-related hospitalization. Pancreatitis burden has continued to increase since 1990, with trends driven by changing exposures to risk factors, population growth, and population aging. We report the national- and state-level estimates of pancreatitis burden. **Methods:** Global Burden of Disease (GBD) is a multinational collaborative research study that quantified the burden of 375 diseases. We reported the most recent incidence, prevalence, mortality, and the age-standardized rates per 100,000 population and the significant change from 1990 to 2023 in the US. Permission was obtained to report state-level estimates. **Results:** In 2023, there were an estimated 235,610 (217,889–251,545) new cases, 403,446 (298,429–514,224) prevalent cases and 5717 (5034–6443) deaths. Pancreatitis contributed 168,062 (147,357–191,446) years of disability-adjusted life years (DALYs). The age-standardized incidence rate (ASIR) was 51.11 (47.33–54.37) and age-standardized mortality rate (ASMR) was 1.05 (0.93–1.19). More burden is noted in men and in the ≥75 age group. High alcohol use contributed to 16.18% (6.14–26.95%) deaths and to 19% (7.16–31.56%) DALYs. Pancreatitis contributed 0.18% (0.16–0.21%) deaths and 0.14% (0.12–0.16) DALYs. From 1990 to 2023, ASIR decreased in all states, ASMR increased in 10 states, and decreased in DC. **Conclusions:** From 1990 to 2023, driven by demographic shifts, the number of cases and deaths have increased. However, when adjusted for age, the overall disease burden remained stable or slightly declined, suggesting that the risk per individual has not worsened. The future burden can be mitigated by specific prevention efforts and by addressing the risk factors.

## 1. Introduction

Pancreatitis is an inflammation of the pancreas occurring due to the early activation of pancreatic enzymes following an insult to the pancreatic tissue and has a significant impact on digestive and endocrine functions. It is classified into two main types: acute and chronic pancreatitis. In acute pancreatitis, noxious stimuli like bile acids and alcohol-derived fatty acid ethyl esters can generate sustained and excessive intracellular Ca^2+^ signals in acinar cells. The resulting Ca^2+^ overload impairs ATP production and, together with premature intracellular activation of digestive enzymes, promotes acinar-cell necrosis, subsequently activating inflammatory pathways that can progress to systemic inflammation and organ failure in severe disease. Acute pancreatitis presents abruptly and chronic pancreatitis has an indolent course, which results from persistent or repeated inflammation [1,2,3].

Several etiologies have been identified, including gallstones, high alcohol consumption, autoimmune conditions, hypertriglyceridemia, genetic disorders, drugs and viral infections, among others. About 35–40% of acute pancreatitis cases are due to gallstones, 30% due to high alcohol use [4], up to 14% due to hypertriglyceridemia [5], and <5% due to medications [6]. Etiology can be identified in about 75% of patients with pancreatitis [3].

The annual incidence of acute pancreatitis varies between 3.4 and 73.4 per 100,000 people. Chronic pancreatitis has an annual incidence rate of 10 per 100,000 and a prevalence of 50 per 100,000 population [3]. Depending on the cause, up to 5.6% of the cases show relapse, with the highest relapse noticed in alcohol-induced pancreatitis [7].

The burden of pancreatitis has increased by 30% in the United States over the past decade. In 2021, it was the third leading cause of hospitalization with a gastrointestinal disease diagnosis, contributing to 1.16 million (9.2%) hospitalizations, 114 emergency department (ED) visits per 100,000 persons, median cost of 7949 USD per visit, and 3441 deaths in the US. About 80% of hospitalizations with pancreatitis are due to mild disease [8]. The increase in pancreatitis burden is unexplained, but can be attributed to increased rates of alcohol use, obesity, and associated gallstones [3].

With socioeconomic development and lifestyle changes, the risk factors for pancreatitis are becoming increasingly diverse [8]. Therefore, a comprehensive analysis of the epidemiological characteristics and dynamic trends of pancreatitis is crucial for developing effective prevention and intervention strategies and to strategize policies that can mitigate its impact through effective prevention and intervention efforts.

This analysis is an update to the previously reported GBD results on the burden of pancreatitis, focusing specifically on national and state-specific data, including District of Columbia (DC), in the US from 1990 to 2023 in both sexes, providing the most recent estimates on incidence, mortality, and DALYs, as they comprehensively represent its burden and help assess the impact. GBD 2023 provides only alcohol-attributable pancreatitis burden estimates, which have been included in this report.

## 2. Materials and Methods

The data for the burden of pancreatitis in the USA from 1990 to 2023 were obtained from the de-identified and publicly available Global Health Data Exchange (GHDx) 2023 results [9]. The GBD 2023 study is developed by collaborative efforts by members throughout the world, and provides population-level estimates for the burden of 375 diseases and 88 risk factors for both males and females in 204 countries and territories from 1990 to 2023. GBD 2023 incorporates newly available data sources and methodological improvements to enhance the reliability of estimates. The GBD results have frequently been refined every 2 to 3 years to estimate mortality and health losses from various diseases, with the most recent being the GBD 2023 update [10]. We utilized GBD 2023 to obtain the standardized definition, estimated datasets, and risk factors for pancreatitis. Permission was obtained from the GBD Secretariat to use the US national and state-specific GBD 2023 data in our study.

Some methodological improvements have been employed to estimate GBD 2023 results, which include: (i) a method for identifying and correcting the estimates displaying excess mortality spikes due to misclassified COVID-19 deaths between the years of 2020 and 2023, (ii) all CODEms that were applied to mortality rates, and (iii) an updated modelling framework for COVID-19 to include the data and preliminary vital reports during the pandemic [10].

The Cause of Death Ensemble model (CODEm) and the Bayesian meta-regression tool DisMod-MR were the main methods used to estimate the datasets for the GBD 2023 study. CODEm evaluates the out-of-sample predictive validity of different statistical models and covariate permutations and combines these results to generate cause-specific estimates. DisMod-MR 2.1 was used to adjust the missing data by applying hierarchical models that used higher-level geographic data. MR-BRT (Meta-Regression—Bayesian, Regularized, Trimmed) was employed to correct the data bias from inconsistent case definitions and measurement methods. Detailed estimation methods for the burden of pancreatitis have been published previously [10,11,12].

GBD 2023 follows the International Classification of Diseases tenth edition (ICD-10) for coding of disease to individually standardize each dataset. Acute pancreatitis is coded as K85, with K85.0 to K85.9 specifying different types and causes, and chronic pancreatitis as K86.0 [13].

In this study, we analyzed the burden of pancreatitis as national and state-specific (including DC) incidence, prevalence, age-standardized incidence rate (ASIR), age-standardized mortality rate (ASMR), disability-adjusted life years (DALYs), age-standardized DALYs rate (ASDR), and by the corresponding 1990–2023 age-standardized average annual rates of change (ARC). These parameters have been disaggregated by sex and age classes (0–14, 15–49, 50–74 and ≥75 years) and have been reported by all causes. All rates were reported per 100,000 person-years, and the GBD global standard population was considered for calculating the age-standardized rates [12]. The number of patients diagnosed with pancreatitis per 100,000 people in a particular year was described as the incidence rate. The mortality rate was defined as the number of deaths per 100,000 people attributable to pancreatitis in a year. The ARC was defined as the percentage change in incidence, prevalence, or mortality rates between the years [14]. The number estimates were rounded to the closest whole number, while the rates, rate changes, and percentages were rounded to two decimal points.

GBD maps each death to a single underlying cause. Pancreatitis-related mortality rates were estimated based on vital registration, verbal autopsy data, and surveillance system data from 1990 to 2023. The GBD 2023 Data Input Sources Tool webpage contains detailed information on the original data sources utilized in this study [15]. The estimates were generated by taking the mean from 1000 draws of the posterior distribution, and uncertainty intervals (UIs) were generated by taking the 2.5th and 97.5th percentile from the model draws This can be interpreted as a 95% probability that the interval contains the true mean or mean change. UI represents the variance in estimation and the uncertainties involved in the data collection process and model selection. A UI overlapping zero includes the possibility that there was no change in burden estimates over time [14].

Using the linear regression models to estimate ARC, temporal trends from 1990 to 2023 were calculated. It was estimated using ordinary least-squares linear regression with calendar year as the independent variable and the age-standardized rates as the dependent variable. The ARC represents the average absolute annual change in the age-standardized rate over the study period (1990–2023) and is expressed as the regression coefficient (β) with its corresponding 95% UI. If ARC and the lower limit of 95% UI were above zero, we considered it an increasing rate. If ARC and the upper limit of 95% UI were below zero, we considered it a decreasing rate. Otherwise, we considered it a stable rate over time [16]. No formal sensitivity analyses were performed as all estimates were obtained from the GBD 2023 database, which incorporates uncertainty estimation through repeated model simulations and reports 95% UI.

GBD 2023 is performed in compliance with GATHER (Guidelines for Accurate and Transparent Health Estimates Reporting) [17]. IRB approval is exempt for the study as we used publicly available de-identified data. The analytic code written to produce this analysis is available in GHDx [18]. GBD analyses were completed using Python (version 3.10.4, Python Software Foundation, Beaverton, OR, USA), Stata (version 13.1, StataCorp, College Station, TX, USA), and R (version 4.4.0, R Foundation for Statistical Computing, Vienna, Austria) [10]. Images were generated on Datawrapper using the GBD 2023 data.

## 3. Results

### 3.1. National Estimates (1990 vs. 2023)

#### 3.1.1. Incidence

In 2023, there were an estimated 235,610 (95% UI: 217,889–251,545) new cases of pancreatitis, 49.1% males and 50.9% females, compared to 184,271 (161,296–207,056) new cases, 46.5% males and 53.4% females, in 1990, or a 27.8% increase. The ASIR for pancreatitis during this period decreased from 63.77 (56.19–71.54) per 100,000 population in 1990 to 51.11 (47.33–54.37) per 100,000 population in 2023. The ARC in ASIR between 1990 and 2023 was −0.20 (−0.27–−0.13) (Table 1). The change in ASIR was statistically significant. The highest incidence rate was noted in ≥75 years age group in 1990 and 2023. A decrease in incidence rate was noted in 15–49 years and 50–74 years age groups for the same duration, but the variation in 0–14 years and ≥75 years age groups was not significant.

#### 3.1.2. Prevalence

In 1990, the prevalence of pancreatitis was estimated to be 320,782 (227,130–422,430), while it was estimated to be 403,446 (298,429–514,224) in 2023. There was a decrease in age-standardized prevalence rate from 1990 to 2023 with an ARC of −0.27 (−0.34–−0.18). The highest prevalence was noted in the 50–74 years age group in 1990 (160,534) and 2023 (233,375).

#### 3.1.3. Mortality

There were 3137 (2723–3574) deaths in 1990 attributed to pancreatitis, of which 54.54% were males and 45.45% were females, compared to 5717 (5034–6443) deaths in 2023, of which 55.84% were males and 44.15% were females. The ASMR for pancreatitis in 1990 was 1.01 (0.88–1.15) compared to 1.05 (0.93–1.19) in 2023 per 100,000 population (Table 2). The ARC in ASMR between 1990 and 2023 was 0.04 (−0.13–0.27). This change in ASMR was not statistically significant. The highest mortality rate was noted in the ≥75 years age group in 1990 and 2023. Although the mortality rate from 1990 to 2023 in all age groups had increased, except for the 0–14 years group, in which it decreased, it was not statistically significant for all groups.

#### 3.1.4. DALYs

The DALYs increased from 104,635 years (88,911–121,162) in 1990 to 168,062 years (147,357–191,446) in 2023 for both sexes (Table 2). The ARC in ASDR was 0.01 (−0.16–0.20) between 1990 and 2023. The highest DALYs rate was noted in the ≥75 years age group in 1990 and 2023. There was a decrease in the DALYs rate in all age groups except for the 15–49 years age group between 1990–2023, in which it was 0.10 (−0.12–0.36), a slight increase but not statistically significant.

### 3.2. State-Wise Including District of Columbia Estimates (1990 vs. 2023)

#### 3.2.1. Incidence

For both sexes, the ASIR was estimated to range from 51.43 (46.01–56.40) in California to 76.73 (65.77–88.10) in West Virginia in 1990, and it continued to be highest in West Virginia at 69.78 (64.53–74.80) but lowest in Oregon at 38.84 (35.75–41.66) in 2023 (Figure 1 and Table 1). Although the least ARC in ASIR between 1990 and 2023 was noted to be in West Virginia at −0.09 (−0.19–0.02), it was not statistically significant. The ARC was found to be statistically significant for the rest of the states and DC. For the same duration, ARC in ASIR was estimated to be lowest in Michigan with −0.10 (−0.16–−0.03) and highest in Oregon with −0.37 (−0.45–−0.27).

#### 3.2.2. Mortality

The ASMR for both sexes was estimated to range from 0.74 (0.62–0.88) in Hawaii to 2.16 (1.82–2.53) in District of Columbia, 1.42 (1.21–1.66) in South Carolina, in 1990, while it ranged from 0.71 (0.62–0.80) in California to 1.75 (1.48–2.05) in West Virginia in 2023 (Figure 2). The ARC in ASMR for both sexes has varied between 1990 and 2023 and was estimated to range from −0.41 (−0.53–−0.25) in DC to 0.37 (0.08–0.74) in West Virginia. There was an increase in ASMR in 10 states (Indiana, Kansas, Kentucky, New Mexico, Oklahoma, Oregon, South Dakota, Tennessee, West Virginia, and Wisconsin) and a decrease in DC.

#### 3.2.3. DALYs

In 1990 the ASDR ranged from 26.80 (22.29–31.16) in Washington to 81.12 (68.89–94.65) in DC and 50.77 (42.65–58.92) in South Carolina, while it ranged from 25.77 (22.71–29.82) in California to 58.78 (50.37–68.77) in West Virginia in 2023 (Figure 3). The ARC in ASDR ranged from −0.45 (−0.57–−0.31) in DC, −0.20 (−0.32–−0.07) in New York to 0.37 (0.09–0.70) in New Mexico between 1990 and 2023, with an increase in New Mexico, Oklahoma, Tennessee, West Virginia, and Wisconsin, and a decrease in New Jersey, New York, and DC.

### 3.3. Burden of Pancreatitis Attributable to High Alcohol Use, Compared to All Causes in the US and Compared to Global Pancreatitis Burden (Table 3)

In the US, high alcohol use contributed to 16.18% (6.14–26.95%) of deaths and 19% (7.16–31.56%) of DALYs due to alcohol-attributed pancreatitis in 2023 in both sexes, compared to 13.83% (5.92–21.13%) of deaths and 17.11% (7.03–26.9%) of DALYs in 1990. In 2023, 22.31% (8.84–36.87%) pancreatitis-related deaths in males were attributable to high alcohol use, while it was only 8.42% (2.72–15.93%) in females. High alcohol use contributed to 24.7% (9.75–40.58%) of pancreatitis-related DALYs in males and 10.47% (3.43–19.62%) in females in 2023.

**Table 3:** Incidence, DALYs and deaths due to pancreatitis in the US compared to global pancreatitis, 1990–2023, both sexes, with annual rate of change between 1990 and 2023.

	1990	2023	1990–2023
	**ASIR per 100,000 persons (95% UI)**	**ASIR per 100,000 persons (95% UI)**	**Annual rate of change for ASIR (95% UI)**
**Global, Pancreatitis**	37.62 (32.20–43.11)	32.91 (28.84–37.17)	−0.13 (−0.14–−0.10)
**US, pancreatitis**	63.77 (56.19–71.54)	51.11 (47.33–54.37)	−0.2 (−0.27–−0.13)
	**ASDR per 100,000 persons (95% UI)**	**ASDR per 100,000 persons (95% UI)**	**Annual rate of change for ASDR (95% UI)**
**Global, Pancreatitis**	61.66 (50.63–75.61)	47.94 (40.57–58.17)	−0.22 (−0.38–0.01)
**US, pancreatitis**	35.75 (30.34–41.47)	35.99 (31.55–41.02)	0.01 (−0.16–0.20)
	**ASMR per 100,000 persons (95% UI)**	**ASMR per 100,000 persons (95% UI)**	**Annual rate of change for ASMR (95% UI)**
**Global, Pancreatitis**	1.76 (1.49–2.16)	1.40 (1.21–1.66)	−0.20 (−0.37–0.03)
**US, pancreatitis**	1.01 (0.88–1.15)	1.05 (0.93–1.19)	0.04 (−0.13–0.27)

The United States all-cause ASMR in both sexes was 681.93 (681.30–682.61) in 1990 and 537.28 (536.81–537.73) in 2023, and all-cause ASDR was 30,422.65 (27,385.26–33,833.77) in 1990 and 27,768.10 (24,608.10–31,264.76) in 2023. Pancreatitis contributed to 0.18% (0.16–0.21%) of the total deaths and 0.14% of DALYs in the US in 2023.

Compared to an estimated global pancreatitis ASIR of 37.62 (32.20–43.11) in 1990 and 32.91 (28.84–37.17) in 2023 with an ARC of −0.13 (−0.14–−0.10), the pancreatitis ASIR in the US is more than twice the global ASIR with a comparable ARC. While the global ASMR was 1.76 (1.49–2.16) in 1990 and 1.40 (1.21–1.66) in 2023, it was 42% and 25% less in the US in the respective years. The US pancreatitis ASDR was almost 50% lower compared to the global ASDR of 61.66 (50.62–75.61) in 1990 and 47.94 (40.57–58.16) in 2023.

We have included the state-specific mortality (Table S1) and DALYs (Table S2) numbers, rate, ASMR, and ASDR with respective ARC data in the supplemental files.

## 4. Discussion

Our study highlights the burden of pancreatitis in the US using the GBD 2023 data. A 27.8% increase in the number of new pancreatitis cases was noted in the US between 1990 and 2023. The estimates reported by GBD 2023 are similar to some of the incidence rates reported previously for acute pancreatitis in the US [19,20,21], but lower compared to the most recently reported numbers of ED diagnoses of pancreatitis (both acute and chronic) in 2021 [8], reflecting the incidence and recurrence of pancreatitis. This difference is also noticed since several sources were utilized in this report, and the possibility of overlapping cases cannot be eliminated [8]. Despite this increase in incidence, the ASIR saw a decline, indicating that population growth and aging drove the rise in cases. This distinction is important because declining age-standardized rates should not be interpreted as a reduction in healthcare burden. A similar trend was noted in gender-specific ASIR. A decrease in prevalence rate was noted between 1990 and 2023, showing an improvement when adjusted for demographic changes. The incidence was consistently high in patients aged 75 years and older, while it has declined in other age groups. The 50–74 years age group had the highest prevalence in both years, reflecting chronic disease accumulation with age. The increase in overall prevalence but decrease in the rate suggests true population increase to be an important contributor to the rising incidence [21].

No change in ASDR was noted (36 per 100,000 in both years) between 1990 and 2023. The highest DALY rates occurred in patients over 75 years of age, depicting severe outcomes in elderly patients. DALYs declined in other age groups except in the 15–49 years group, in whom a slight but non-significant increase was observed. The individual changes in years lived with disability (YLDs) and years of life lost (YLL) contribute to the overall DALYs. It is expected to increase in the next two decades [16]. The divergence between stable age-standardized disability rates and increasing absolute DALYs further emphasizes the influence of demographic changes on disease burden.

Pancreatitis-related deaths increased by 82.2% between 1990 and 2023, with more deaths noted in men compared to women. Despite this increase, the ASMR remained stable, increasing insignificantly from 1.01 to 1.05 per 100,000 during this period. Patients aged 75 years and older had the highest mortality. The death rates slightly increased across all age groups except in the 0–14 years group, where a decrease was noted. These trends in mortality are similar to the previously reported data [8,21], but lower than that reported by Lo, et al. [22]. Although the stable age-standardized mortality rate likely reflects improvements in diagnosis, critical care, minimally invasive interventions, nutritional support, and multidisciplinary management of pancreatitis, these improvements have been offset at the population level by demographic aging and population growth. Consequently, the absolute number of pancreatitis-related deaths has continued to increase despite advances in clinical care [21]. Prognosis is particularly poor in the elderly age group, with up to 20% mortality rate for severe acute pancreatitis [23]. This could be due to the presence of comorbidities in the elderly contributing to the overall prognosis. The increase in the absolute number of pancreatitis-related deaths despite relatively stable ASMR likely reflects demographic changes rather than worsening disease outcomes. Population growth and aging have increased the number of individuals at risk, particularly among older adults who experience greater disease severity and mortality. In addition, improved survival from other chronic diseases has resulted in a larger elderly population susceptible to pancreatitis.

Across the US states, pancreatitis incidence showed substantial geographic variation. Although most states saw statistically significant declines in ASIR between 1990 and 2023, West Virginia’s small decrease was not significant, indicating a persistently high burden there. The steepest decline occurred in Oregon, suggesting notable progress in reducing pancreatitis incidence. Prevalence patterns mirrored population size. California consistently had the highest number of pancreatitis cases. This reflects both population differences and broader demographic trends rather than major shifts in disease risk by state. California consistently reported the largest number of prevalent cases, which is expected given its large population size rather than increased disease risk [24]. The DALYs also varied across states, suggesting that although national averages have remained stable, some states, especially in the Appalachian and southern regions, continue to face a disproportionately high pancreatitis burden. Mortality rates varied widely across states with West Virginia recording the highest mortality rate, and California the lowest. Ten states saw rising mortality, while the District of Columbia experienced a decline, suggesting regional differences in disease management and prevention effectiveness.

West Virginia consistently demonstrated one of the highest pancreatitis mortality and DALY rates, but is one of the states with the highest obesity prevalence [25], which is a major risk factor for gallstones, but has the lowest alcohol consumption [24]. The relative contribution of obesity, alcohol consumption, gallstone disease, healthcare access, and socioeconomic factors cannot be determined. The observed geographic differences may reflect variation in exposure to the established risk factors, healthcare access, socioeconomic conditions, coding practices, or other unmeasured regional factors. However, these potential contributors should be interpreted as hypotheses rather than causal explanations. Improving access to preventive healthcare, specialist referral, and early management of pancreatitis may also contribute to reducing regional disparities, and the effectiveness of these interventions at the state level was not evaluated in the present study. These observations should therefore be interpreted as hypotheses that warrant further investigation rather than demonstrated causal associations.

High alcohol use is an important contributing factor to the burden of pancreatitis [4,26]. In the US, high alcohol use accounted for 16.2% of deaths and 19% of DALYs from alcohol-attributable pancreatitis in 2023, which has significantly increased from 1990. The burden was higher in males (22.3% of deaths, 24.7% of DALYs) than in females (8.4% and 10.5%, respectively). A similar difference was observed in a study published by Hauksson, et al. [27], observing a higher burden of alcohol-attributed pancreatitis in males. This difference can be explained due to high consumption rates of alcohol in males and the physiological differences wherein males are known to be at higher risk of developing pancreatitis compared to females [28,29,30], with hazard ratio of 1.63 [30], contributing to the overall burden of pancreatitis. Although all-cause mortality and DALY rates have declined since 1990 in the US, alcohol-related pancreatitis remains a persistent contributor, responsible for about 0.18% of total deaths in 2023. Reducing alcohol-attributable pancreatitis requires a comprehensive public health approach. Evidence-based strategies include routine alcohol screening and brief interventions in primary care, improved access to behavioral counseling and addiction treatment services, taxation and pricing policies, restrictions on alcohol availability and marketing, and public education regarding harmful alcohol consumption.

Compared to global figures, the US has more than double the incidence rate of pancreatitis but roughly half the mortality and disability burden, reflecting better management and healthcare outcomes despite ongoing risks from high alcohol consumption [28]. The global yearly incidence and mortality rates are comparable to the previously published estimates [31,32]. Compared with international analyses, the United States continues to demonstrate substantially higher incidence but lower mortality, likely reflecting differences in healthcare access, early diagnosis, and availability of advanced pancreatic care. This discrepancy suggests that improvements in clinical management may have reduced case fatality without substantially reducing disease occurrence.

Several factors may explain the decline in age-standardized pancreatitis burden but increased absolute burden from 1990 to 2023. These observations illustrate an epidemiological transition in pancreatitis. Although age-standardized incidence and disability have declined modestly, the absolute burden continues to rise because demographic changes outweigh improvements in age-specific disease risk. Advances in diagnostic and treatment modalities have improved diagnosis, management, and outcomes for pancreatitis. Serum lipase and imaging techniques including endoscopic ultrasound and magnetic resonance cholangiopancreatography enable earlier and more accurate diagnosis. Supportive care and early enteral nutrition with the availability of minimally invasive procedures for complications have reduced severe outcomes in acute pancreatitis while enzyme therapy and pain control have improved care in chronic pancreatitis [3,19]. Additionally, public health efforts targeting alcohol, tobacco, obesity, and diabetes have indirectly lowered the age-standardized rates of pancreatitis despite no targeted initiatives being available for pancreatitis. Newer medications may also help mitigate the burden in the future. An important development that may influence the future burden of acute pancreatitis is the emergence of therapies directed at its underlying cellular mechanisms. Zegocractin is an investigational inhibitor of calcium release-activated calcium (CRAC) channels that limits Orai1-mediated Ca^2+^ entry. In a randomized, placebo-controlled phase 2b trial of patients with acute pancreatitis and systemic inflammatory response syndrome, zegocractin did not significantly improve the primary endpoint of time to solid-food tolerance in the overall study population. However, dose-related benefits were observed in prespecified higher-risk subgroups, and several secondary outcomes favored treatment, including new-onset severe respiratory failure and time to medically indicated discharge [33]. If efficacy and safety are confirmed in larger trials, such treatment could reduce severe acute complications, recurrent pancreatic injury and reduce the risk of progression to chronic pancreatitis. The burden of pancreatitis is expected to rise in the next 15 years [16]. Forecasts suggest that the burden of pancreatitis will increase primarily because of population growth and population aging rather than increasing age-specific disease risk. In addition, the rising prevalence of some of the risk factors may further contribute to future disease burden. Specific strategies should be established for effective prevention or early treatment of pancreatitis.

### Limitations

This study, although comprehensive, has several limitations that should be considered when interpreting the results. The data used in this analysis is sourced from national health records that may vary in accuracy and completeness, leading to underreporting or misclassification. This analysis does not account for the health-care cost data, which is important for understanding the broader societal impact due to pancreatitis. As with the previous estimates, GBD 2023 also reports both acute and chronic pancreatitis as a single cause, generalizing the burden of two different conditions. Combining these distinct disorders into a single category may obscure important differences in disease burden and temporal trends. Furthermore, the burden attributable to recurrent acute pancreatitis progressing to chronic pancreatitis cannot be adequately assessed. Separate estimation of acute, chronic, and recurrent pancreatitis in future GBD iterations would provide a more accurate assessment of their individual global burden and improve the utility of these data for clinicians, researchers, and policymakers. GBD 2023 reports only the pancreatitis burden attributable to high alcohol use due to data constraints, reducing the scope to understand the other potential risk factors. Self-reported data, especially risk factors like alcohol consumption, are prone to biases, including underreporting, which could affect the accuracy of attributable burden estimates. Although the present study identifies substantial geographic heterogeneity across U.S. states, it was not designed to formally evaluate spatial clustering or quantify the contribution of other determinants.

To overcome these limitations, future research should focus on improving datasets by integrating comprehensive national health surveys to generate population-specific data. Furthermore, it is important to account for differences in prevention and treatment approaches influenced by socioeconomic factors to produce more precise and contextually relevant estimates. Addressing these issues will help establish accurate and effective strategies to address pancreatitis.

## 5. Conclusions

From 1990 to 2023, the number of pancreatitis cases and deaths have increased, largely driven by demographic shifts. However, when adjusted for age, the incidence, prevalence, mortality, and overall disease burden remained stable or slightly declined, suggesting that the risk of pancreatitis per individual has not worsened. Elderly populations continue to bear the greatest impact, while improvements in healthcare may have mitigated more severe outcomes. These findings emphasize the need for prevention strategies targeting established and potentially modifiable causes of pancreatitis, including harmful alcohol use, smoking, gallstone disease, hypertriglyceridemia, and other metabolic risk factors. Early identification and treatment of the underlying etiology after an episode of acute pancreatitis may also reduce recurrent attacks and their subsequent health burden.

Most U.S. states saw slight decline in pancreatitis incidence and disease burden rates after adjusting for age, but substantial geographic disparities persist. West Virginia consistently had the highest rates of incidence and mortality, while western states like Oregon and California showed the lowest and greatest improvements. These variations likely reflect differences in alcohol use, healthcare access, socioeconomic conditions, and preventive health behaviors across regions; however, due to limited data on the risk factors, this study was not designed to determine the relative contribution of these variables. Persistent geographic disparities further emphasize the need for region-specific prevention strategies targeting modifiable risk factors. Future burden estimates should distinguish acute from chronic pancreatitis and addressing risk factors to better inform disease-specific prevention, healthcare planning, and resource allocation.

## Figures and Tables

**Figure 1:**
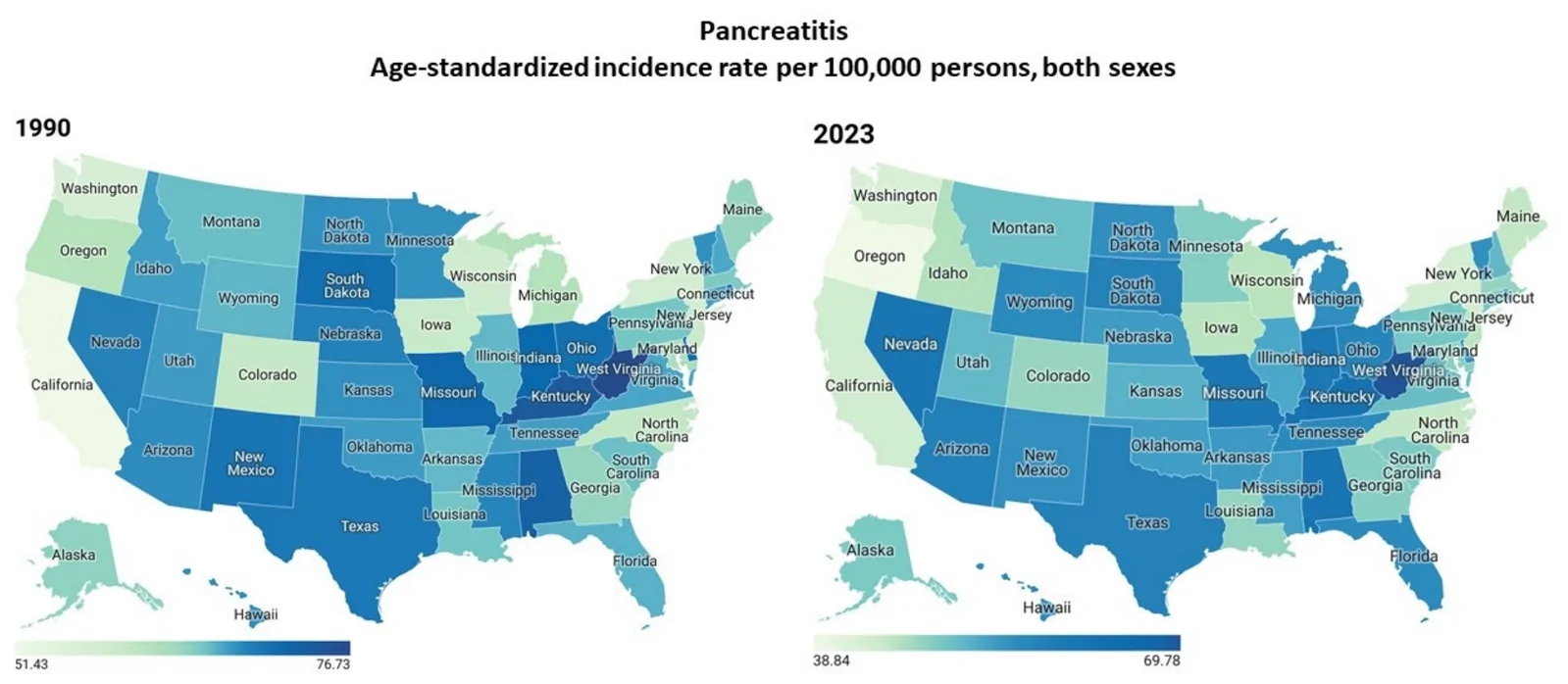
Age-standardized incidence rate per 100,000 population for pancreatitis in the United States, 1990–2023, both sexes.

**Figure 2:**
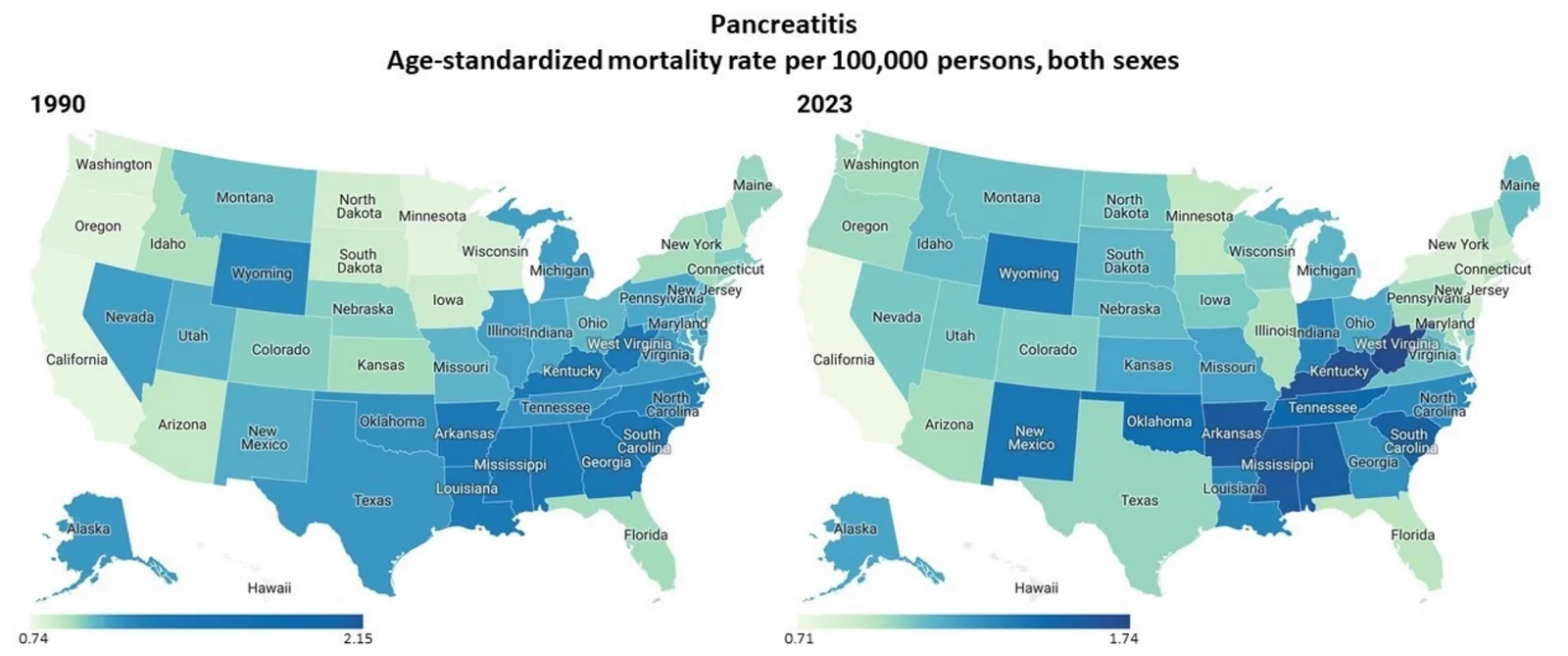
Age-standardized mortality rate per 100,000 population for pancreatitis in the United States, 1990–2023, both sexes.

**Figure 3:**
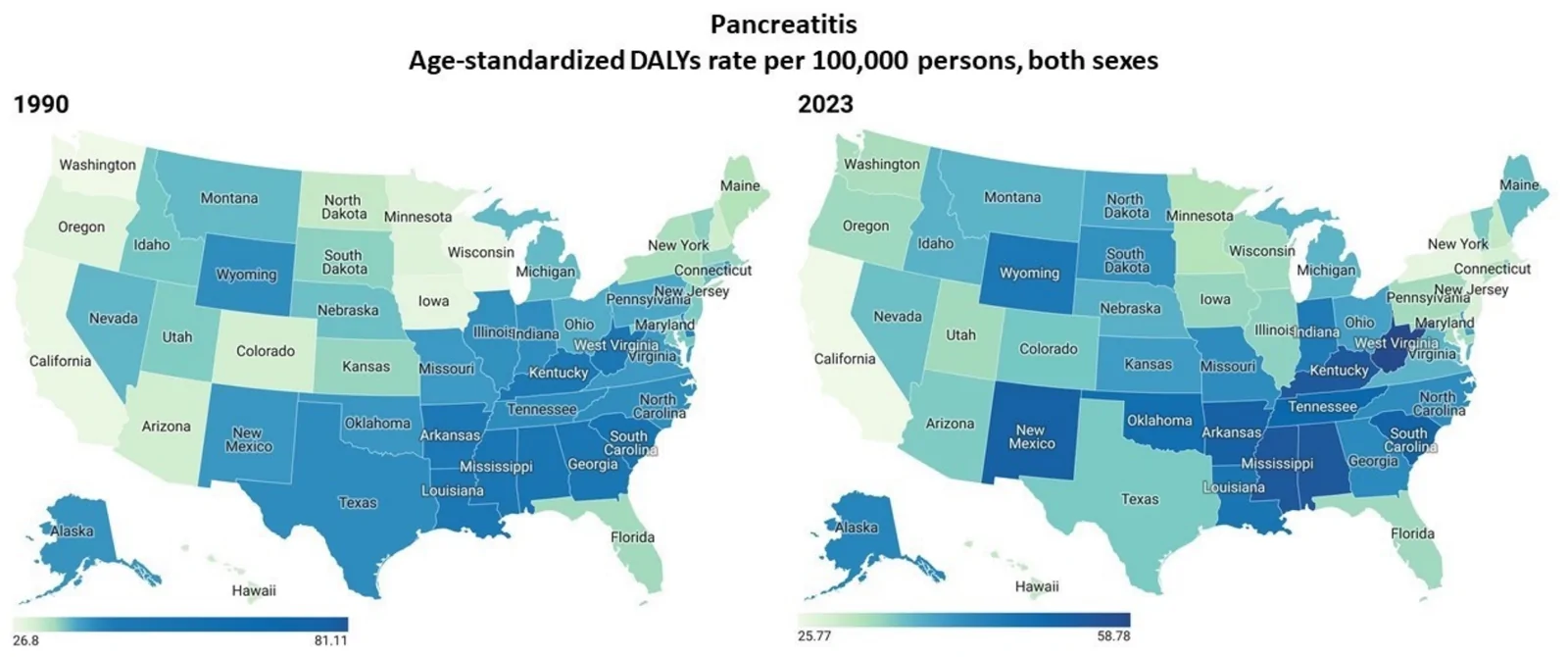
Age-standardized DALYs rate per 100,000 population for pancreatitis in the United States, 1990–2023, both sexes.

**Table 1:** Incidence of pancreatitis in the US, 1990–2023, both sexes, with annual rate of change between 1990 and 2023.

Pancreatitis	1990			2023			1990–2023	
	Cases (95% UI)	Rate per 100,000 (95% UI)	ASIR per 100,000 (95% UI)	Cases (95% UI)	Rate per 100,000 (95% UI)	ASIR per 100,000 (95% UI)	Annual Rate of Change for Incidence Rate (95% UI)	Annual Rate of Change for ASIR (95% UI)
National Estimates								
United States of America	184,271 (161,296–207,056)	72.53 (63.49–81.5)	63.77 (56.19–71.54)	235,610 (217,889–251,545)	70.24 (64.96–75)	51.11 (47.33–54.37)	−0.04 (−0.12–0.07)	−0.20 (−0.27–−0.13)
Male	85,751 (75,036–96,907)	69.10 (60.46–78.09)	64.03 (56.15–72.15)	115,898 (106,819–123,516)	69.71 (64.25–74.29)	51.72 (47.79–55.12)	0.01 (−0.08–0.11)	−0.19 (−0.26–−0.11)
Female	98,519 (86,161–110,353)	75.80 (66.29–84.91)	63.45 (55.99–70.89)	119,712 (110,675–127,951)	70.77 (65.43–75.64)	50.63 (46.82–53.86)	−0.07 (−0.15–0.03)	−0.20 (−0.26–−0.13)
State Estimates								
Alabama	3436 (2944–3934)	83.24 (71.33–95.31)	72.68 (62.47–83.32)	4309 (3972–4642)	83.72 (77.18–90.2)	61.26 (56.54–65.77)	0.01 (−0.11–0.15)	−0.16 (−0.24–−0.05)
Alaska	335 (277–403)	59.45 (49.12–71.6)	64.62 (54.44–75.72)	445 (385–500)	62.68 (54.15–70.45)	49.35 (43.11–54.18)	0.05 (−0.12–0.25)	−0.24 (−0.31–−0.15)
Arizona	2867 (2512–3233)	76.35 (66.88–86.09)	68.17 (60.06–76.68)	5917 (5507–6294)	79.89 (74.36–84.99)	58.03 (53.94–61.77)	0.05 (−0.04–0.15)	−0.15 (−0.22–−0.07)
Arkansas	1899 (1655–2173)	79.05 (68.88–90.44)	66.46 (58.6–76.01)	2238 (2074–2368)	72.62 (67.31–76.85)	54.3 (50.4–57.5)	−0.08 (−0.17–0.03)	−0.18 (−0.25–−0.09)
California	16,817 (15,053–18,629)	55.22 (49.43–61.17)	51.43 (46.01–56.4)	22,933 (21,250–24,454)	58.93 (54.61–62.84)	43.57 (40.26–46.5)	0.07 (−0.01–0.14)	−0.15 (−0.20–−0.10)
Colorado	2209 (1916–2531)	65.5 (56.81–75.06)	59.93 (52.68–67.72)	3729 (3463–3944)	63.44 (58.92–67.1)	47.68 (44.45–50.36)	−0.04 (−0.13–0.09)	−0.20 (−0.28–−0.12)
Connecticut	2690 (2292–3098)	80.41 (68.53–92.63)	67.37 (57.96–77.73)	2602 (2385–2810)	71.12 (65.18–76.8)	49.99 (45.84–53.34)	−0.12 (−0.22–0.01)	−0.26 (−0.34–−0.15)
Delaware	549 (467–636)	80.49 (68.51–93.19)	70.97 (60.64–82.45)	827 (757–890)	80 (73.28–86.12)	55.86 (51.46–59.41)	−0.01 (−0.13–0.14)	−0.21 (−0.30–−0.11)
District of Columbia	462 (386–547)	74.97 (62.51–88.76)	62.89 (52.71–75.16)	384 (328–434)	58.79 (50.29–66.45)	46.26 (39.72–51.83)	−0.22 (−0.28–−0.14)	−0.26 (−0.33–−0.19)
Florida	11,227 (9928–12,547)	84.66 (74.87–94.62)	66.71 (58.97–75.09)	19,061 (17,627–20,383)	84.58 (78.22–90.45)	56.21 (52.2–59.96)	−0.01 (−0.08–0.08)	−0.16 (−0.23–−0.09)
Georgia	4576 (3984–5166)	68.95 (60.03–77.85)	64.41 (56.29–72.57)	7009 (6426–7526)	63.7 (58.41–68.4)	48.63 (44.86–51.88)	−0.08 (−0.15–0.02)	−0.24 (−0.30–−0.17)
Hawaii	861 (730–1004)	75.95 (64.39–88.59)	67.95 (57.73–79.27)	1109 (967–1246)	79.87 (69.65–89.68)	56.27 (48.64–64.47)	0.05 (−0.03–0.16)	−0.17 (−0.22–−0.12)
Idaho	748 (630–866)	72.51 (61.03–83.9)	67.42 (57.11–78.39)	1199 (1095–1295)	60.53 (55.27–65.37)	46.22 (42.25–49.51)	−0.17 (−0.26–−0.03)	−0.31 (−0.39–−0.21)
Illinois	8815 (7743–9915)	75.55 (66.36–84.98)	66.5 (58.53–74.65)	9313 (8529–9992)	73.76 (67.56–79.14)	52.98 (48.85–56.46)	−0.02 (−0.10–0.07)	−0.20 (−0.27–−0.13)
Indiana	4605 (4014–5168)	81.34 (70.91–91.29)	71.63 (62.55–80.21)	5502 (5038–5906)	79.56 (72.85–85.4)	59.42 (54.79–63.59)	−0.02 (−0.11–0.09)	−0.17 (−0.24–−0.09)
Iowa	2016 (1750–2305)	71.21 (61.82–81.44)	58.83 (51.43–66.91)	2055 (1903–2182)	63.68 (58.95–67.6)	45.81 (42.32–48.58)	−0.11 (−0.19–−0.01)	−0.22 (−0.29–−0.13)
Kansas	1972 (1671–2259)	78.13 (66.2–89.49)	67.88 (58.38–78.03)	2040 (1873–2199)	69.43 (63.74–74.84)	51.98 (47.92–55.51)	−0.11 (−0.21–0.02)	−0.23 (−0.32–−0.12)
Kentucky	3186 (2743–3586)	84.6 (72.85–95.23)	73.95 (63.99–83.31)	3869 (3560–4138)	85.24 (78.44–91.17)	62.25 (57.62–66.38)	0.01 (−0.09–0.13)	−0.16 (−0.24–−0.06)
Louisiana	3044 (2592–3492)	70.72 (60.21–81.15)	65.71 (56.28–75.61)	2987 (2747–3203)	65.33 (60.1–70.06)	47.88 (44.35–51.13)	−0.08 (−0.18–0.05)	−0.27 (−0.35–−0.17)
Maine	943 (794–1094)	75.33 (63.41–87.33)	64.3 (54.39–75.28)	973 (888–1054)	69.51 (63.46–75.26)	45.28 (41.41–48.34)	−0.08 (−0.18–0.07)	−0.3 (−0.37–−0.20)
Maryland	3448 (2996–3918)	70.63 (61.38–80.27)	62.49 (54.87–70.55)	4141 (3838–4394)	67.16 (62.24–71.26)	48.49 (45.02–51.33)	−0.05 (−0.15–0.05)	−0.22 (−0.29–−0.14)
Massachusetts	4672 (4121–5211)	76.32 (67.32–85.12)	65.25 (57.55–72.63)	4780 (4428–5096)	67.78 (62.79–72.26)	47.92 (44.52–51.03)	−0.11 (−0.17–−0.03)	−0.27 (−0.32–−0.20)
Michigan	6575 (5851–7311)	69.36 (61.73–77.12)	61.63 (54.79–68.12)	8112 (7503–8635)	80.09 (74.08–85.25)	55.51 (51.4–59)	0.15 (0.07–0.25)	−0.1 (−0.16–−0.03)
Minnesota	3415 (2864–3943)	76.49 (64.13–88.31)	67.84 (57.82–78.48)	3894 (3561–4195)	67.35 (61.59–72.55)	48.84 (44.81–52.3)	−0.12 (−0.22–0.02)	−0.28 (−0.36–−0.18)
Mississippi	1979 (1713–2236)	75.28 (65.17–85.07)	68.72 (59.87–77.72)	2138 (1980–2294)	72.83 (67.45–78.12)	53.76 (49.78–57.56)	−0.03 (−0.11–0.07)	−0.22 (−0.28–−0.14)
Missouri	4392 (3775–4996)	84.13 (72.32–95.7)	71.89 (61.99–81.76)	5108 (4702–5490)	82.21 (75.67–88.36)	60.11 (55.53–63.93)	−0.02 (−0.11–0.1)	−0.16 (−0.24–−0.07)
Montana	622 (520–711)	76.23 (63.74–87.12)	66.25 (56–76.57)	812 (735–885)	71.33 (64.6–77.72)	50.66 (46.14–54.59)	−0.06 (−0.17–0.07)	−0.24 (−0.32–−0.14)
Nebraska	1284 (1093–1477)	79.72 (67.84–91.75)	69.05 (59.18–79.78)	1420 (1299–1543)	71.38 (65.29–77.58)	54.12 (49.49–58.27)	−0.10 (−0.2–0.02)	−0.22 (−0.3–−0.12)
Nevada	955 (829–1091)	76.75 (66.64–87.71)	68.99 (60.06–78.33)	2633 (2446–2821)	82.06 (76.24–87.94)	60.73 (56.59–64.64)	0.07 (−0.04–0.19)	−0.12 (−0.20–−0.02)
New Hampshire	857 (726–998)	75.69 (64.17–88.16)	67.09 (57.37–77.69)	1074 (984–1165)	76.28 (69.9–82.75)	51.38 (47.29–55.13)	0.01 (−0.11–0.16)	−0.23 (−0.32–−0.13)
New Jersey	5511 (4843–6160)	69.77 (61.31–77.99)	58.52 (51.7–65.35)	6077 (5616–6492)	64.44 (59.55–68.84)	45.66 (42.35–48.86)	−0.08 (−0.15–0.01)	−0.22 (−0.28–−0.15)
New Mexico	1158 (978–1336)	74.66 (63.05–86.16)	70.19 (59.74–80.7)	1632 (1500–1757)	77.17 (70.91–83.06)	55.34 (51.09–59.13)	0.03 (−0.08–0.21)	−0.21 (−0.3–−0.09)
New York	12,323 (10,846–13,825)	67.23 (59.17–75.42)	56.95 (50.37–63.77)	12,369 (11,512–13,181)	62.5 (58.17–66.61)	43.05 (40.01–45.79)	−0.07 (−0.14–0.02)	−0.24 (−0.30–−0.17)
North Carolina	4665 (4095–5267)	68.74 (60.34–77.6)	60.13 (52.85–67.39)	6674 (6163–7144)	62.48 (57.69–66.87)	45.2 (42.03–48.3)	−0.09 (−0.17–−0.01)	−0.25 (−0.31–−0.18)
North Dakota	507 (425–584)	78.01 (65.38–89.95)	67.89 (57.15–78.97)	564 (491–635)	72.26 (62.88–81.39)	55.52 (48.45–61.26)	−0.07 (−0.15–0.01)	−0.18 (−0.25–−0.11)
Ohio	8985 (7826–10,056)	81.35 (70.86–91.05)	70.7 (61.73–79.04)	9657 (8860–10,277)	81.25 (74.54–86.46)	57.33 (52.93–61)	−0.01 (−0.09–0.10)	−0.19 (−0.25–−0.11)
Oklahoma	2498 (2142–2852)	77.86 (66.75–88.9)	67.47 (58.21–77.12)	2847 (2628–3051)	70.09 (64.71–75.13)	53.92 (49.68–57.94)	−0.1 (−0.19–0.02)	−0.20 (−0.29–−0.09)
Oregon	2098 (1748–2434)	71.98 (59.96–83.49)	61.43 (51.62–71.34)	2416 (2202–2636)	56.63 (51.63–61.8)	38.84 (35.75–41.66)	−0.21 (−0.31–−0.08)	−0.37 (−0.45–−0.27)
Pennsylvania	9686 (8424–10,894)	80.05 (69.62–90.03)	65.62 (57.36–73.93)	9306 (8573–10,009)	71.32 (65.7–76.71)	49.69 (45.94–52.92)	−0.11 (−0.19–−0.02)	−0.24 (−0.30–−0.16)
Rhode Island	857 (721–983)	83.72 (70.44–96.12)	70.3 (59.73–80.64)	851 (765–932)	76.63 (68.87–83.94)	53.59 (48.39–58.1)	−0.08 (−0.19–0.03)	−0.24 (−0.32–−0.14)
South Carolina	2599 (2210–2961)	72.87 (61.97–83.03)	66.21 (56.67–75.72)	3663 (3341–3933)	68.89 (62.84–73.97)	49.23 (45.26–52.57)	−0.05 (−0.15–0.08)	−0.26 (−0.33–−0.16)
South Dakota	575 (487–662)	80.96 (68.66–93.29)	70.88 (60.76–81.89)	709 (628–782)	77.37 (68.55–85.36)	57.82 (51.91–62.78)	−0.04 (−0.13–0.06)	−0.18 (−0.26–−0.09)
Tennessee	3890 (3364–4408)	77.94 (67.41–88.32)	67.39 (58.25–76.08)	5471 (5031–5843)	76.5 (70.35–81.71)	56.28 (52.14–60.07)	−0.02 (−0.11–0.09)	−0.16 (−0.23–−0.09)
Texas	12,661 (11,045–14,092)	72.81 (63.51–81.03)	69.62 (60.92–77.99)	21,559 (19,971–22,925)	70.89 (65.67–75.38)	57.89 (53.8–61.36)	−0.03 (−0.11–0.06)	−0.17 (−0.23–−0.09)
Utah	1100 (924–1272)	62.26 (52.3–72.03)	67.46 (57.2–78.72)	2036 (1860–2180)	58.94 (53.85–63.12)	51.28 (46.98–54.83)	−0.05 (−0.17–0.09)	−0.24 (−0.33–−0.13)
Vermont	442 (370–510)	76.83 (64.38–88.78)	67.85 (57.62–78.28)	535 (475–593)	82.04 (72.82–90.95)	55.13 (49.21–60.54)	0.07 (−0.04–0.21)	−0.19 (−0.26–−0.1)
Virginia	4738 (4068–5394)	74.91 (64.32–85.29)	67.16 (58.02–76.32)	6017 (5533–6459)	69.84 (64.22–74.97)	50.89 (46.92–54.4)	−0.07 (−0.16–0.05)	−0.24 (−0.31–−0.16)
Washington	3178 (2747–3624)	63.64 (55.01–72.58)	56.5 (48.86–64.13)	4512 (4188–4797)	57.77 (53.62–61.43)	42.24 (39.4–45.01)	−0.09 (−0.18–0.02)	−0.25 (−0.32–−0.17)
West Virginia	1703 (1450–1943)	93.28 (79.41–106.42)	76.73 (65.77–88.1)	1832 (1684–1972)	102.44 (94.16–110.27)	69.78 (64.53–74.8)	0.10 (−0.02–0.25)	−0.09 (−0.19–0.02)
Wisconsin	3338 (2906–3786)	66.9 (58.24–75.89)	57.93 (50.78–65.71)	3849 (3579–4106)	64.63 (60.09–68.94)	45.88 (42.62–48.78)	−0.03 (−0.12–0.07)	−0.21 (−0.27–−0.12)
Wyoming	331 (278–386)	71.58 (59.98–83.49)	66.59 (56.77–78.07)	449 (399–496)	76.93 (68.32–84.99)	56.26 (50.55–60.98)	0.07 (−0.05–0.21)	−0.16 (−0.24–−0.06)

ASIR: Age-standardized incidence rate; UI: uncertainty interval.

**Table 2:** DALYs and deaths due to pancreatitis in the US, 1990–2023, both sexes, with annual rate of change between 1990 and 2023.

Pancreatitis	1990		2023		1990–2023
	DALYs				
**United States of America**	**DALYs (95% UI)**	**ASDR per 100,000 persons (95% UI)**	**DALYs (95% UI)**	**ASDR per 100,000 persons (95% UI)**	**Annual rate of change for ASDR (95% UI)**
Both Sexes	104,635 (88,911–121,162)	35.75 (30.34–41.47)	168,062 (147,357–191, 446)	35.99 (31.55–41.02)	0.01 (−0.16–0.20)
Male	60,895 (50,595–71,727)	45.27 (37.59–53.32)	100,704 (84,697–116,687)	45.49 (38.06–53.07)	0.00 (−0.22–0.27)
Female	43,740 (35,969–53,081)	27.17 (22.12–33.21)	67,358 (54,181–81,129)	26.59 (21.64–32.15)	−0.02 (−0.22–0.20)
	**Deaths**				
**United States of America**	**Deaths (95% UI)**	**ASMR per 100,000 persons (95% UI)**	**Deaths (95% UI)**	**ASMR per 100,000 persons (95% UI)**	**Annual rate of change for ASMR (95% UI)**
Both Sexes	3137 (2723–3574)	1.01 (0.88–1.15)	5717 (5034–6443)	1.05 (0.93–1.19)	0.04 (−0.13–0.27)
Male	1711 (1443–2035)	1.30 (1.10–1.55)	3193 (2708–3739)	1.31 (1.10–1.52)	0.00 (−0.22–0.31)
Female	1426 (1180–1704)	0.76 (0.62–0.91)	2525 (1989–3076)	0.80 (0.63–0.97)	0.05 (−0.21–0.32)

## Data Availability

The data used in this study is publicly available and can be accessed through the Global Health Data Exchange (GBDx) 2023 [9].

## Supplementary Materials

The following supporting information can be downloaded at: https://www.mdpi.com/article/10.3390/jcm15197497/s1, Table S1: Mortality of pancreatitis in the US, 1990–2023, both sexes, with annual rate of change between 1990 and 2023; Table S2: Disability-adjusted life years (DALYs) of pancreatitis in the US, 1990–2023, both sexes, with annual rate of change between 1990 and 2023.
